# Ecosystem Scale Declines in Elk Recruitment and Population Growth with Wolf Colonization: A Before-After-Control-Impact Approach

**DOI:** 10.1371/journal.pone.0102330

**Published:** 2014-07-16

**Authors:** David Christianson, Scott Creel

**Affiliations:** 1 School of Natural Resources and the Environment, University of Arizona, Tucson, Arizona, United States of America; 2 Department of Ecology, Montana State University, Bozeman, Montana, United States of America; Point Blue Conservation Science, United States of America

## Abstract

The reintroduction of wolves (*Canis lupus*) to Yellowstone provided the unusual opportunity for a quasi-experimental test of the effects of wolf predation on their primary prey (elk – *Cervus elaphus*) in a system where top-down, bottom-up, and abiotic forces on prey population dynamics were closely and consistently monitored before and after reintroduction. Here, we examined data from 33 years for 12 elk population segments spread across southwestern Montana and northwestern Wyoming in a large scale before-after-control-impact analysis of the effects of wolves on elk recruitment and population dynamics. Recruitment, as measured by the midwinter juvenile∶female ratio, was a strong determinant of elk dynamics, and declined by 35% in elk herds colonized by wolves as annual population growth shifted from increasing to decreasing. Negative effects of population density and winter severity on recruitment, long recognized as important for elk dynamics, were detected in uncolonized elk herds and in wolf-colonized elk herds prior to wolf colonization, but not after wolf colonization. Growing season precipitation and harvest had no detectable effect on recruitment in either wolf treatment or colonization period, although harvest rates of juveniles∶females declined by 37% in wolf-colonized herds. Even if it is assumed that mortality due to predation is completely additive, liberal estimates of wolf predation rates on juvenile elk could explain no more than 52% of the total decline in juvenile∶female ratios in wolf-colonized herds, after accounting for the effects of other limiting factors. Collectively, these long-term, large-scale patterns align well with prior studies that have reported substantial decrease in elk numbers immediately after wolf recolonization, relatively weak additive effects of direct wolf predation on elk survival, and decreased reproduction and recruitment with exposure to predation risk from wolves.

## Introduction

The extent that top-down forces from predators, bottom-up forces from resources, and abiotic forces such as weather interact to affect population growth in prey species is one of the most central questions in ecology [1]–[3]. In large herbivores, the role of top-down and bottom-up forces is of particular interest because large herbivores often have high connectivity within food webs and strong influences on lower trophic levels over large geographic areas [4]–[6]. Elk (*Cervus elaphus*) in the Greater Yellowstone Ecosystem (GYE) are well-recognized as a case study of bottom-up, top-down, and abiotic forcing in a keystone species due to the maintenance of long-term monitoring programs, which allow comparison of elk population dynamics before and after the reintroduction of a top predator, the grey wolf (*Canis lupus*).

Prior to the reintroduction of wolves to the GYE in 1995 and 1996, over five decades of research and monitoring of Yellowstone elk dynamics consistently found that (1) variation in juvenile recruitment explained most of the annual variation in elk population growth and that (2) negative density dependence and winter severity were the strongest drivers of annual variation in recruitment [7]–[11]. These patterns are commonly seen in other ecosystems, and in other long-lived iteroparous species [12]–[14]. In the GYE however, elk numbers declined by >50% after wolf reintroduction in some areas [15], [16], exceeding the 5–30% declines predicted by models of wolf-elk dynamics [17]. This discrepancy prompted more recent analyses of GYE elk population dynamics that have incorporated density dependence and winter severity, but also considered recent data on predation rates by wolves, other climate variables, and harvest rates from elk hunting. Surprisingly, these more recent studies have concluded that direct killing by wolves had relatively weak effects on elk population growth [18], [19] due to (1) low absolute rates of wolf predation on elk [20], [21], and (2) low reproductive value of wolf-killed elk [15], [19], [22], [23]. Offtake from hunters, particularly of reproductive females, exceeded offtake by wolves in the years immediately following reintroduction and consequently hunting has been suggested as a factor possibly explaining declining elk abundance following wolf reintroduction [15], [18], [19], [23].

Decreased survival of female elk due to wolves and human hunting is an interesting result by itself because adult survival is often observed (or assumed) to be high and relatively invariant in elk and other long-lived, iteroparous species [12], [14], [23]. Just as intriguing, these more recent analyses of elk dynamics challenge the pre-wolf consensus that negative density dependence and winter severity were the primary drivers of population dynamics. For example, Eberhardt et al [19] considered snowpack and summer precipitation over the pre-wolf/post-wolf period and found no ‘useful relationships’ between these variables and population growth. Vucetich et al. [18] reexamined pre-wolf elk dynamics and found that hunting and growing season precipitation had effects on annual growth rates 2–4 times larger than the effects of winter snowfall and elk density.

**Figure 1:**
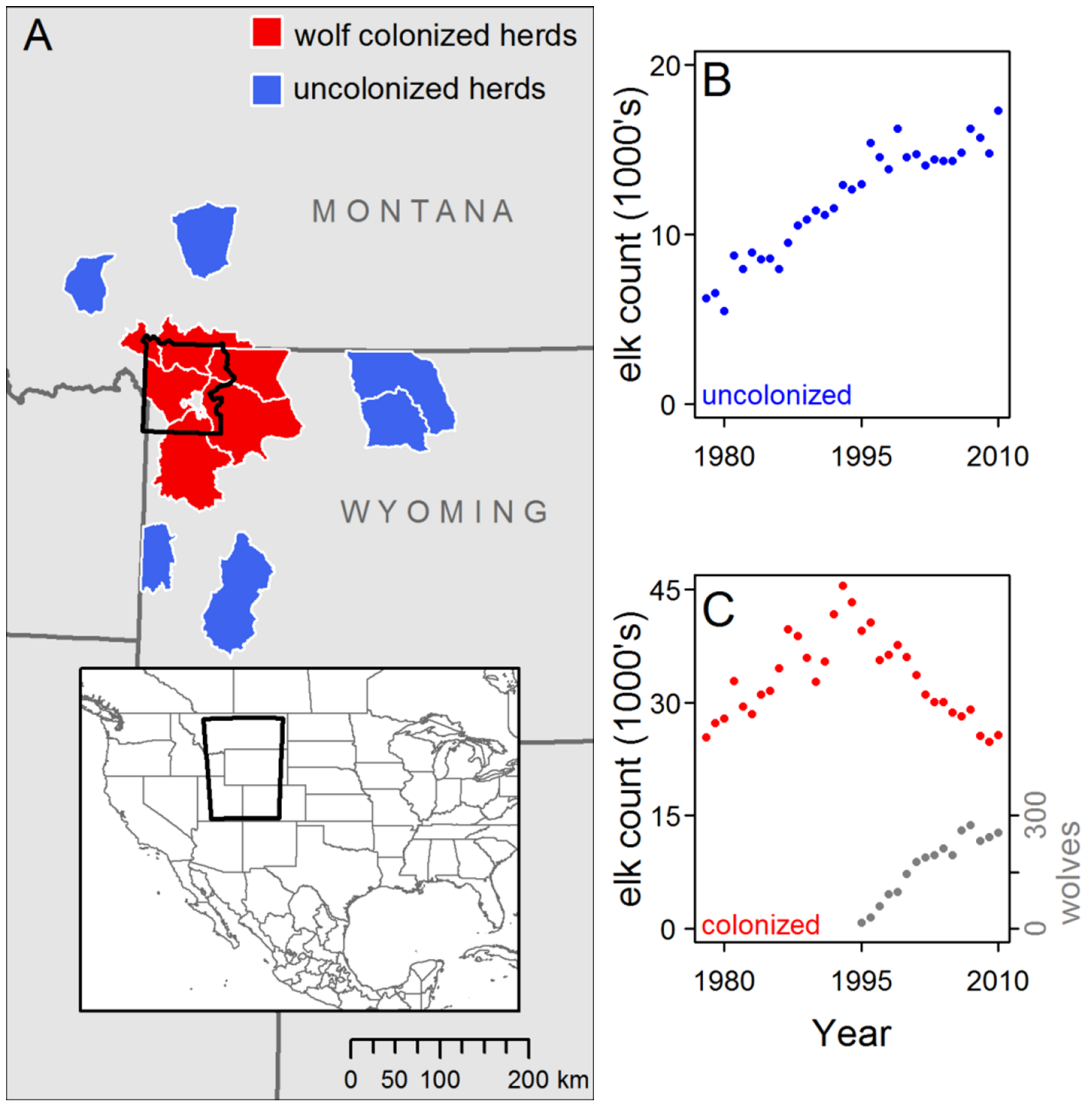
Population trends and distributions of 12 elk herds in the Greater Yellowstone Ecosystem. (A) Annual ranges of six elk herds colonized by wolves after wolf reintroduction to Yellowstone National Park (YNP, black polygon) in 1995 and 1996 and six elk herds that were not known to be recolonized by wolves before 2010. Areas between polygons also contain elk herds (including herds that also migrate into or near YNP in summer) but with complex histories of wolf colonization and were excluded from this study. Grey polygon in center of YNP is Yellowstone Lake. (B) Counts of elk summed across uncolonized herds from 1978–2010. (C) Counts of elk summed across herds colonized by wolves from 1978–2010, which represent the majority of the Greater Yellowstone Ecosystem elk population. In uncolonized herds summed trend counts clearly illustrate the predominant trends (B), although these herds do not likely belong to a single interconnected elk population. Demographic data were split between two periods, 1978–1995 and 1996–2010, in all herds to examine the effects of wolf recolonization on juvenile∶female ratios using a before-after control impact approach.

The contrast between recent results and prior research is curious, as Eberhardt et al. [19] also noted. Contrary to the expectation of population dynamics controlled mainly by negative density-dependence in juvenile recruitment, recruitment has apparently declined despite greatly reduced elk density since wolf reintroduction [15], [16] (Fig. 1). What is now driving declining recruitment and the apparent negation of well-documented effects of density dependence and winter severity since the reintroduction of an apex predator? These are important questions that remain unresolved for one of the best-studied ecosystems in the scientific literature.

Here, we compiled data on the factors known or hypothesized to influence elk recruitment for a 33-yr data set from >40,000 elk, using a before-after-control-impact design with six herds in the GYE recolonized by wolves and six herds that were not colonized by wolves. This approach reduces (but does not completely eliminate) the problem of interpreting concurrent changes in several limiting factors, which has hampered inferences from studies of single population segments. We tested whether density dependence and winter severity continued to influence elk recruitment after wolf reintroduction in the same manner as reported by pre-wolf studies [7], [9]–[11]. We tested an emerging hypothesis that decreased growing season precipitation may have depressed elk recruitment since wolf reintroduction [24], [25]. While it has not been hypothesized that hunting might explain declines in recruitment, several studies have reported strong effects of hunting on elk population dynamics [15], [18], [19], so we also tested whether changes in hunting since wolf reintroduction contributed to recruitment declines. The spatiotemporal scale and resolution of available data did not allow us to include grizzly bear (*Ursus arctos*) numbers in formal statistical tests, but we used available data to evaluate the hypothesis that changes in bear density might have caused the observed changes in elk recruitment and dynamics.

## Methods

### Data sources and compilation

We compiled 33 years (1978–2010) of demographic and count data from 12 elk herds in northwestern Wyoming and southwestern Montana, USA. Six of these herds were colonized by wolves following reintroductions to Yellowstone National Park (YNP) in 1995 and 1996, and six herds were not known to be colonized before 2010, for a complete before-after-control-impact (BACI) study designed to estimate the total effect of wolves on elk recruitment rates (396 herd-years representing ∼40,000 elk/year). Because herds could not be randomly assigned to treatment (wolf-colonized) and control (uncolonized), we used covariates to account for the possibility that other limiting factors abruptly shifted at the time of wolf recolonization, only in the herds colonized by wolves. We selected the six elk herds at the core of the GYE with annual ranges overlapping YNP that were re-exposed to wolves by 1997 and continuously occupied by wolves thereafter (‘wolf-colonized herds’). We identified the six control herds (uncolonized herds) nearest to the GYE with no known resident wolf packs before 2010; only two uncolonized herds with adequate data were found in Montana (and no herds in Idaho), so we selected the four elk herds in Wyoming closest to the GYE that met the criteria for being ‘wolf-free’ for a balanced design of six treatment herds and six control herds. The six control herds are not known to overlap at any time of year.

We compiled annual wolf, elk, and climate data beginning on June 1 in each year, at the peak of parturition [20]. For each herd-year, we compiled autumn harvest rates, midwinter herd counts, and midwinter demographic composition surveys conducted by Montana Fish Wildlife and Parks (MFWP) and Wyoming Game and Fish Department (WGFD) and filed in annual reports with the U.S. Fish and Wildlife Service in compliance with the Pittman-Robertson Federal Aid in Wildlife Restoration Act. We compiled additional data for elk herds primarily within YNP from published articles with YNP data [11], [19], [23], [26], public communications of YNP research (www.nps.gov/yell/parknews) and personal communication with scientists monitoring YNP elk (P. Cross, Northern Yellowstone Cooperative Wildlife Working Group, U.S. Geological Survey, Bozeman, Montana). Herd counts and demographic surveys were always conducted in winter, primarily via aerial transects over the winter range of each herd, but search effort, transect lengths, or sightability were rarely recorded (see Discussion). We excluded data from one herd-year with an exceptionally small classification survey (57 animals). We also excluded the juvenile∶female ratio for Yellowstone's Northern Range herd in 1988, when a severe winter followed unprecedented wildfires that burned >40% of this herd's annual range. Annual elk harvest figures were collected by MFWP and WGFD via telephone and mail surveys of elk hunting license holders, corrected for response rate.

### Wolf pack sizes and distribution

Wolves were reintroduced in 1995 and 1996 to Yellowstone National Park, so we defined pre-wolf (1978–1995) and post-wolf (1996–2010) periods in the data set (for simplicity, we also refer to the latter period in uncolonized herds as “post-wolf” even though wolves never occupied these areas). Wolves were closely monitored by the USFWS throughout the post-wolf period, and pack sizes and kernel home range estimates or pack centroids were compiled annually in federal reports by USFWS and NPS in compliance with the Northern Rocky Mountain Wolf Recovery Plan (available at www.fws.gov/mountain-prairie/species/mammals/wolf and www.nps.gov/yell/naturescience/wolfrpts.htm). With these data we could classify each elk herd as being colonized or uncolonized by wolves for each year during the post-wolf period and estimate the number of wolves annually overlapping each herd's range.

### Winter severity and growing season precipitation

We compiled weather records from 84 National Resource Conservation Service (U.S. Department of Agriculture) SNOTEL stations and Snow Course transects located within the boundaries of each elk herd's annual range. We tabulated end-of-winter snowpack (for the winter just preceding parturition) in each herd-year as the annual mean snow-water equivalents from April 23 to May 8 (‘May 1 snowpack’) at each SNOTEL station or Snow Course transect. We standardized these values by subtracting the site-specific mean and dividing by the site-specific standard deviation, then averaged the standardized May 1 snowpack across all monitoring sites within a herd's range (each herd's range included 2–17 snowpack monitoring sites). For each SNOTEL station, we also averaged standardized annual cumulative precipitation falling May 1 through September 30 across stations within a herd's annual range as a measure of growing season precipitation in each herd-year.

### Statistical Analysis

#### Assessing the accuracy of juvenile∶female ratios

**Figure 2:**
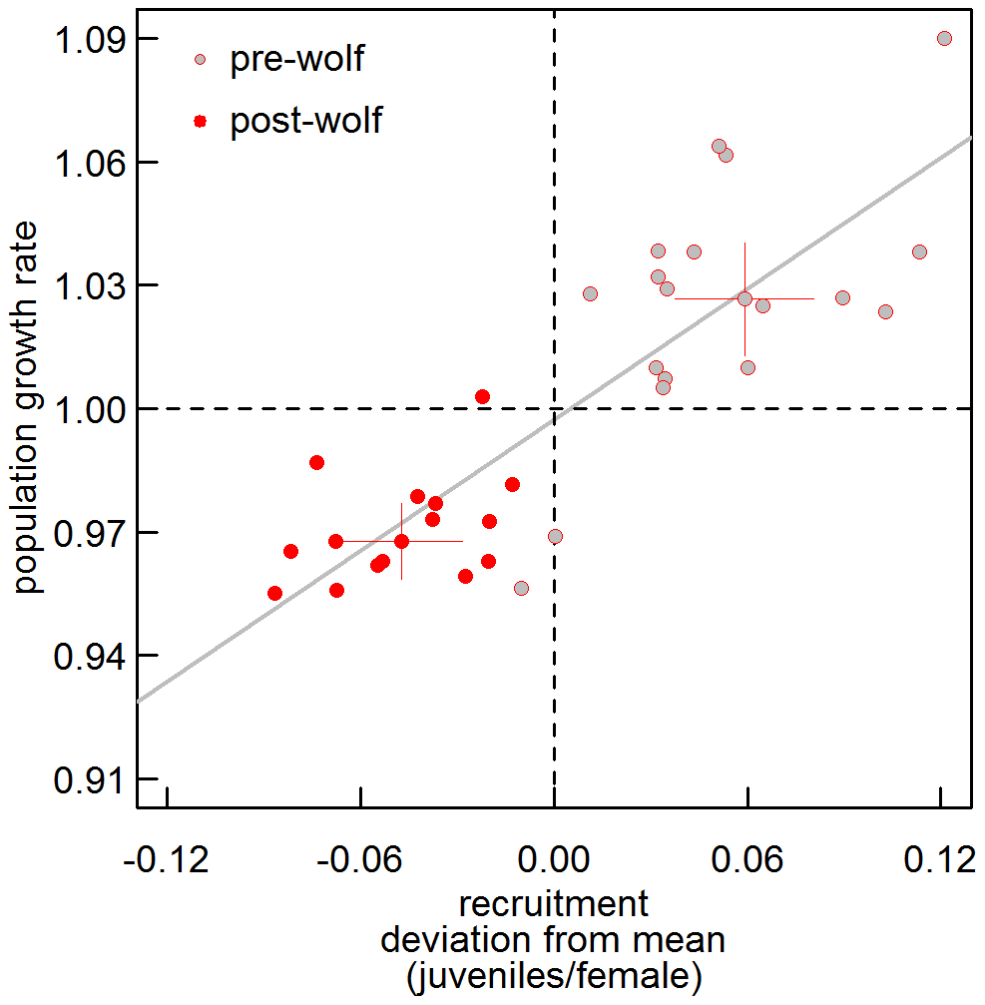
Population growth rate and recruitment of elk (5-yr localized gemoetric means and 5-yr localized means, respectively) in the Greater Yellowstone Ecosystem 1978 to 2010 for six herds recolonized by wolves. Whiskered points are means and 95% CI for the pre-wolf (1978–1995) and post wolf (1996–2010) period. Recruitment is expressed as absolute deviation from 33-yr herd-specific means.

While not a true measure of absolute recruitment of juveniles into the breeding population, juvenile∶female ratios from midwinter surveys have been shown to correlate strongly with juvenile survival and population growth rates [11], [14], which we also confirmed here (see Fig. 2 below). Juvenile to female ratios have been criticized as unreliable because the detection rate of calves accompanying marked females can vary considerably throughout the year [27]. Here, we avoided this effect by comparing midwinter juvenile∶female ratios that were collected at approximately the same time each year, and by using data from surveys that counted all calves, whether with their mothers or not. Unbiased and relatively precise estimates of the juvenile∶female ratio can be obtained by classifying a few hundred individuals with random sampling. Sampling strategies were not recorded for most the surveys compiled here, and error may have also been introduced through misclassification of age-sex classes [28]. Sampling bias and misclassification error, if important in these data, would tend to inflate variance and mask effects on juvenile∶female ratios (increasing the likelihood of type II errors), which we considered when interpreting our results. Using linear regression, we tested whether the coefficient of variation in annual juvenile∶female ratios was negatively correlated with the average number of animals classified (both as an absolute number and as the equivalent proportion of the annual count) as both misclassification and sampling error, if important, would have stronger effects at smaller sample sizes. We censored one herd from this analysis because this herd was surveyed differently than the other 11 herds, using repeated sampling of groups over several weeks in winter, i.e., double sampling was inherent and unavoidable with this sampling design, thus the number of unique individuals classified was unknown [29]. However, we found no evidence that sample size influenced variation in annual juvenile∶female ratios whether this herd was included or excluded from this analysis (see Results). Finally, we tested whether juvenile∶female ratios predicted subsequent changes in herd demography as cohorts aged. Most (76%) classification surveys also classified males ∼21 months of age (in addition to juveniles, females, and adult males), as ‘yearling’ males can be differentiated in midwinter from males ≥2 years using their distinctive ‘spike’ antler shape. Classification error should be highest for yearling males owing to their mixed affinity for both female and male groups [30] and relatively small antlers. We tested whether the yearling male∶female ratio correlated positively with the previous year's juvenile∶female ratio (when yearling males were juveniles) after an adjustment for the number of harvested yearling males and adult females between surveys.

#### Indexing elk density from counts

Herd counts and classification surveys were often conducted separately, and no count accompanied the classification survey in 15.4% of herd-years. To model the effects of density on juvenile∶female ratios we standardized elk counts within each herd as an index of elk density and we replaced missing counts with interpolation between available counts (*loess* function, R Development Core Team 2012) with a maximum gap of four years between recorded counts – most (70.8%) gaps in counts were ≤2 years. We did not include any remaining herd-years with missing counts (i.e., herd-years within count gaps >4 years) or missing classification surveys in our models for effects on recruitment. However, for the purpose of showing general, ecosystem-wide population trends and for illustrating the importance of recruitment responses for population dynamics (see Results and Discussion), we replaced the remaining missing counts (10.1% of herd-years) using interpolation with a maximum gap of nine years – even in this extreme case, the direction of the interpolated population trend was confirmed by a parallel trends in harvest.

#### Assessing variation and trend in juvenile∶female ratios and relationship with population growth

We first performed a simple paired t-test to test whether herd-mean juvenile∶female ratios differed between pre-wolf and post-wolf periods in each treatment (n = 6) and to describe the general trend in juvenile∶female ratios. To test whether juvenile∶female ratios correlated with population growth, we estimated lambda (λ) using year-over-year ratios of uncorrected elk counts summed across the six colonized elk herds (which overlapped part of the year). To reduce the effects of sampling and interpolation error [31], we used 5-year localized geometric means of lambda (moving window averages) and tested for a correlation with 5-yr localized means of the juvenile∶female ratio [31]. Pooling data in a similar fashion for uncolonized herds was less biologically meaningful because these herds likely did not interact or overlap, but we fit the same relationship in uncolonized herds to test whether the broad correlation between population growth and recruitment was consistent across treatments.

#### Testing for effects of harvest on juvenile∶female ratios

We first tested whether hunting may have influenced recruitment by examining the correlation between the number of juveniles harvested per female and the subsequent juvenile∶female ratio of the herd observed in winter. Large harvest ratios of juveniles∶female should have a negative effect on midwinter juvenile∶female ratios. A negative correlation between the two would suggest that increased juvenile harvest is one cause of decreased midwinter recruitment ratios. A positive correlation would suggest midwinter juvenile∶female ratios are largely established before hunting begins and hunting has little effect. For 75% of 396 herd-years, we were able to compile data on the demographic composition of harvests (some annual reports were incomplete and one elk herd was never hunted). We tested the strength and direction of the relationship between harvest and herd juvenile∶female ratios using linear regression. Finally, we tested whether age-sex structure and harvest of female and juvenile elk (as a percent of total counts) changed after wolf reintroduction. Initial tests found no effect of harvest on ratios. However, we assumed sampling units (herd-years) were not independent, allowing for only six degrees of freedom, which may have overestimated the variance and increased the risk of committing Type II errors. We confirmed no statistically significant effect of harvest by conducting the same analyses assuming all herd-years were independent, i.e., without restricting our degrees of freedom (see Results).

#### Testing for effects of wolf colonization on juvenile∶female ratios using mixed-effects modelling

After confirming a positive relationship between juvenile∶female ratios and population growth and testing for effects of harvest (see Results), we analyzed the entire 33-yr data set as a BACI quasi-experiment by fitting a linear mixed effects model using restricted maximum likelihood to estimate the effect of wolf reintroduction using the *lme4* package in R (R Core Team, 2012). This model directly estimated the main effects and interaction between site (wolf-colonized herds vs. uncolonized herds) and treatment (pre-colonization, 1978–1995 vs. post-colonization, 1996–2010), with a random effect (on the intercept) of herd identity nested within treatments and a random effect (on the intercept) of year nested within period to account for unmeasured differences among replicates and to avoid pseudoreplication by accounting for repeated annual measurements within each herd. With this design, a statistically significant interaction between site and treatment is strong evidence for an effect of wolf colonization on elk recruitment.

#### Estimating effects of changes in density and climate with wolf colonization using linear modelling

A few observations with high leverage can drive results in a BACI model, especially given a small set of observations in each conditional state. The mixed effects model structure above is also inefficient for quantifying inconsistent effects of density or climate factors across treatments or periods. We used weighted-mean regression coefficients from fitting separate regressions within each herd to describe temporal trends in recruitment and to estimate effect sizes of elk density, late winter snowpack, and growing season precipitation [32] in the pre-wolf period (12 regressions, one for each herd), post-wolf period (12 regressions) and full time series (12 regressions).Negative density dependence is often measured as a negative correlation between annual population growth rate (lambda) and population density, but bias and sampling error in counts can bias both mean and variance estimates of density-dependent effects [31]. Our approach avoids this problem by measuring the effect of elk abundance on the juvenile∶female ratio, which has previously been shown to be strongly correlated with population growth [11], [14], a correlation that we confirmed for these data (see Results).

Finally, before fitting herd-specific models of recruitment on density, snowpack, and precipitation, we centered juvenile∶female ratios on herd-specific means (i.e. annual ratios were restated as differences from 1978–2010 herd averages) and we standardized density, snowpack and precipitation (i.e. each metric was restated in terms of herd-specific standard deviations from its mean) so that coefficient estimates from the linear model would be directly comparable. Because the six wolf-colonized herds at the core of the ecosystem overlapped to varying degrees in summer when herds collectively migrated towards Yellowstone National Park [20], [33] we considered whether elk density in these herds was better indexed by standardizing the pooled annual counts (Fig. 1C) before fitting models of juvenile∶female ratios. Standardized pooled counts produced better fit than individual herd counts for wolf-colonized herds and were used in our analysis (assessed from Q-Q plots, residual plots on predicted values, and adjusted r-squared values). We examined the separate linear regression for each herd to confirm that patterns apparent at the ecosystem level were consistent with patterns within herds; in every case they were (with the exception that the weighted regression coefficient indicated a weak 9% decline in juvenile∶female ratios in uncolonized herds, but declines were not detectable within any single uncolonized herd, see Results). To minimize the likelihood of committing type I errors in these secondary tests (due to partial dependence among annual observations), we restricted our degrees of freedom to the number of wolf-colonized (n = 6) and uncolonized herds (n = 6) within a treatment or pre-wolf (n = 18) and post-wolf (n = 15) years within a time-period. This restriction yields conservative tests.

#### Observed versus expected recruitment after wolf reintroduction using linear modelling

After confirming that growing season precipitation had no effect on juvenile∶female ratios (see Results), we used coefficients for elk abundance and late winter snowpack from linear models fit to *pre-wolf* (1978–1995) data to predict the expected juvenile∶female ratio in each *post-wolf* year (1996–2010) given the elk numbers and winter snowpack observed after wolf reintroduction in each herd, as in Garrott et al. 2009 [26]. We then compared observed to expected juvenile∶female ratios. In wolf-colonized herds after colonization, we also restated these ratios in terms of absolute number of juveniles within the count to better illustrate effects on population dynamics. To do this, mean juvenile∶female ratios (or mean responses in the ratio) were multiplied by the product of the female proportion of the classification survey and the count in each herd-year. We cautiously interpret this metric as juveniles within counts, not within populations, (a distinction with critical implications, see Discussion). When estimating absolute numbers in this fashion, we substituted missing data on the proportion of females within a herd (no classification count was available for 8% of post-wolf, colonized herd-years) with herd-specific means. The female proportion of herds was largely invariant in the post-wolf period (mean ±95% CI: 0.684±0.015) and substituting missing values with means had little effect on our results, e.g., excluding herd-years with missing demographic data produced a <1% difference in the estimated effect of wolf predation on total juvenile abundance (see Results and Discussion).

#### Estimating the effect of direct wolf predation on post-wolf juvenile∶female ratios

Finally, traditional approaches to predator-prey dynamics in ungulate populations would assume that the most likely cause for any observed decline in the juvenile∶female ratio after reintroduction is decreased juvenile survival due to wolf predation (wolves killing juveniles before they can be counted in midwinter surveys). Following the same approach used by many prior studies of this system [15], [20], [21], [23], [26], [34] we estimated the contribution of wolf predation to declining juvenile∶female ratios by estimating total offtake of juveniles in each herd-year. The spatial distribution of wolf packs and the number of wolves in each pack were closely monitored and recorded annually as part of the wolf recovery and monitoring program, providing estimates of total ‘wolf-days’ in each herd. Seasonal variation in rates of wolf predation in juvenile elk (juveniles/wolf/day) have been estimated by direct observation of several GYE wolf packs, overlapping this study spatially and temporally [35]. We combined these estimates of wolf numbers and daily kill-rates (elk/wolf/day) to estimate juvenile offtake in each herd-year prior to midwinter classification surveys: specifically, total wolf offtake of juveniles, *P*, in herd *h* in year *y* can be estimated as:
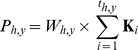
where *W* is the number of wolves overlapping with herd *h*'s annual range in year *y* and **K** is a vector of seasonally variable, daily kill rates spanning the biological year with the 1^st^ element being the kill rate on 1 June (peak of parturition) and element *t_h,y_* being the kill rate on the day of the composition survey in that herd-year. We derived the vector of daily kill rates by linear interpolation between Metz et al's [35] estimates for daily kill rates during their four sampling periods: early winter, late winter, spring and summer. With these estimates for total offtake in each herd year, we examined what proportion of the difference between observed and expected juvenile∶female ratios might be explained by direct wolf predation.

## Results

### Assessing the accuracy of juvenile∶female ratios

A total of 815 112 elk were classified in the 12 herds between 1978 and 2010. The total number of individuals classified averaged 18 493 elk (±2559 95% CI) annually in six herds colonized by wolves, and 6 207 elk (±569 95% CI) annually in six herds uncolonized by wolves. The mean number of individuals classified as a proportion of the total number counted in each herd was 0.864 (±0.094 95% CI). We found no evidence for a negative relationship between the coefficient of variation in annual juvenile∶female ratio and mean number of animals classified (coefficient ± SE, −0.000006±0.000014, F_1,9_ = 0.218, P = 0.652) or mean proportion of the annual count classified (−0.006±0.192, F_1,9_ = 0.00, P = 0.975) suggesting that variation in demographic ratios was not strongly influenced by sampling error or misclassification error. Linear regression of the yearling male∶female ratio onto the prior year's juvenile∶female ratio (after adjusting yearling male∶female counts for offtake by hunters between surveys) revealed a strong correlation (F_1,233_ = 119, P<0.001, r^2^ = 0.338) with an intercept no different from 0 (intercept ± SE, −0.030±0.018, P = 0.0899) and a slope near 0.5 (0.531±0.049, P<0.001), consistent with generally low non-harvest mortality in juvenile elk between midwinter counts and approximate unity in juvenile sex ratios [23], [36], further confirming that classification counts were unbiased. Despite these results, it is likely that sampling error and misclassification of individuals inflated variation in juvenile∶female ratios to some degree. This sampling error, as well as potential misclassification of herd-years to wolf-treatment or period, would only be expected to obscure effects of wolves on recruitment (rather than creating spurious effects), nevertheless we detected several effects and differences, as reported below.

### Variation and trend in juvenile∶female ratios and relationship with population growth

Mean juvenile∶female ratios in the six elk herds colonized by wolves declined by 35% from 0.297 juveniles∶female to 0.193 juveniles∶female after wolf reintroduction (or −0.104 juveniles/female ±0.039, 95% CI, Welch's paired t = 6.82, df = 5, P = 0.001). Notably, declines in juvenile∶female ratios occurred between the pre- and post-wolf periods in all six colonized herds, and were statistically significant in all but one (P = 0.106), suggesting a causal limiting factor that operated consistently throughout the ecosystem. This decline is equivalent to 2 193 (±152, 95% CI) juveniles ‘missing’ annually from combined herd counts in wolf-colonized herds (which averaged a combined 21 088 female elk [±1462, 95% CI] annually) after wolf reintroduction. Juvenile∶female ratios in neighboring elk herds, uncolonized by wolves, declined by 9% over the same period (or −0.034 juveniles/female ±0.006, 95% CI, Welch's paired t = 13.74, df = 5, P<0.001), although declines were not statistically significant (P≥0.067) within any single uncolonized herd.

Uncolonized elk herds were at relatively low densities in the pre-wolf period (compare scales of y-axes in Figs. 1B and 1C) and elk counts were higher in uncolonized herds after wolf reintroduction (Welch's t_23.7_ = 9.10, P<0.001, Fig. 1B). In contrast, there was no difference in counts in wolf-colonized herds between periods (Welch's t_31_ = 1.58, P = 0.125, Fig. 1C) but this was the result of positive population growth from low to high densities in in the pre-wolf period (geometric mean λ ±95% CI: 1.026±0.014) and negative population growth from high to low densities in the post-wolf period (0.968±0.009 Fig. 2). In uncolonized herds, growth was positive in pre-wolf years (1.052±0.022) and post-wolf years (1.008±0.013). Importantly, the juvenile∶female ratio was strongly correlated with population growth, in both wolf-colonized (Fig. 2, slope coefficient ± SE: 0.530±0.061, F_1,31_ = 74.55, P<0.001, r^2^ = 0.706) and uncolonized herds (0.812±0.20, F_1,31_ = 16.42, P<0.001, r^2^ = 0.346) confirming the relevance of midwinter juvenile∶female ratios to population dynamics.

### Assessing the effect of harvest on juvenile∶female ratios

We found no evidence that changes in hunting drove the observed changes in juvenile∶female ratios after wolf reintroduction in wolf-colonized herds. The proportion of ‘antlerless’ (juvenile and female) elk harvested by hunters averaged 0.138 (±0.019 95% CI) of the number of elk counted in each herd. Harvests averaged 0.218 juveniles per female (±0.070 95% CI) prior to wolf reintroduction versus 0.137 (±0.080 95% CI) after wolf reintroduction (Welch's *t* for the difference_7.9_ = 2.14, P = 0.065), a 37% decline in harvest of juveniles∶female from wolf-colonized herds. We found no evidence for a negative relationship between autumn harvest ratios and midwinter herd ratios either before (slope coefficient ± SE, 0.10±0.20, F_1,58_ = 0.28 P = 0.613) or after wolf reintroduction (0.23±0.18, F_1,61_ = 1.22, P = 0.227). A positive correlation between autumn harvest ratios and midwinter herd ratios was evident in the full time series (0.37±0.10, F_1,121_ = 3.70, P<0.001). This positive correlation and the similarity between a 37% decline in harvest ratios of juveniles∶female and the observed 35% decline in midwinter ratios after wolf reintroduction suggests that annual declines in recruitment were largely established before autumn, when most harvests occurred. This comparison of pre- and post-wolf harvest ratios excludes data from the Madison-Firehole elk herd, where elk were not hunted, yet juvenile∶female ratios declined by 50% after wolf reintroduction [29]. Harvest ratios in uncolonized herds were of similar magnitude to those of colonized herds (0.187±0.040, 95% CI) but did not change over the 33-year period (Welch's t_8.69_ = 0.10, P = 0.924). All of these results consistently suggest that human harvest cannot explain the observed regional trends in juvenile∶female ratios (and indeed would be expected to produce changes opposite to those observed).

### Effects of wolf colonization on juvenile∶female ratios using mixed-effects modelling

A linear mixed-effects model treating the complete data set as a BACI design estimated that the effect of wolf reintroduction was −0.073 juveniles/female (site×treatment effect, ±0.014 SE, P = 0.006), the equivalent of a 24.6% decline from the pre-wolf ratio of 0.297 juveniles∶female. To our knowledge, this is the broadest and most direct test of changes in elk recruitment attributable to wolf recolonization to date. However, the model structure does not allow for efficient estimation of coefficients for other limiting factors, nor does it allow for these effects to vary between treatments or periods.

### Testing for simultaneous effects of density and climate with wolf colonization using linear modelling

**Figure 3:**
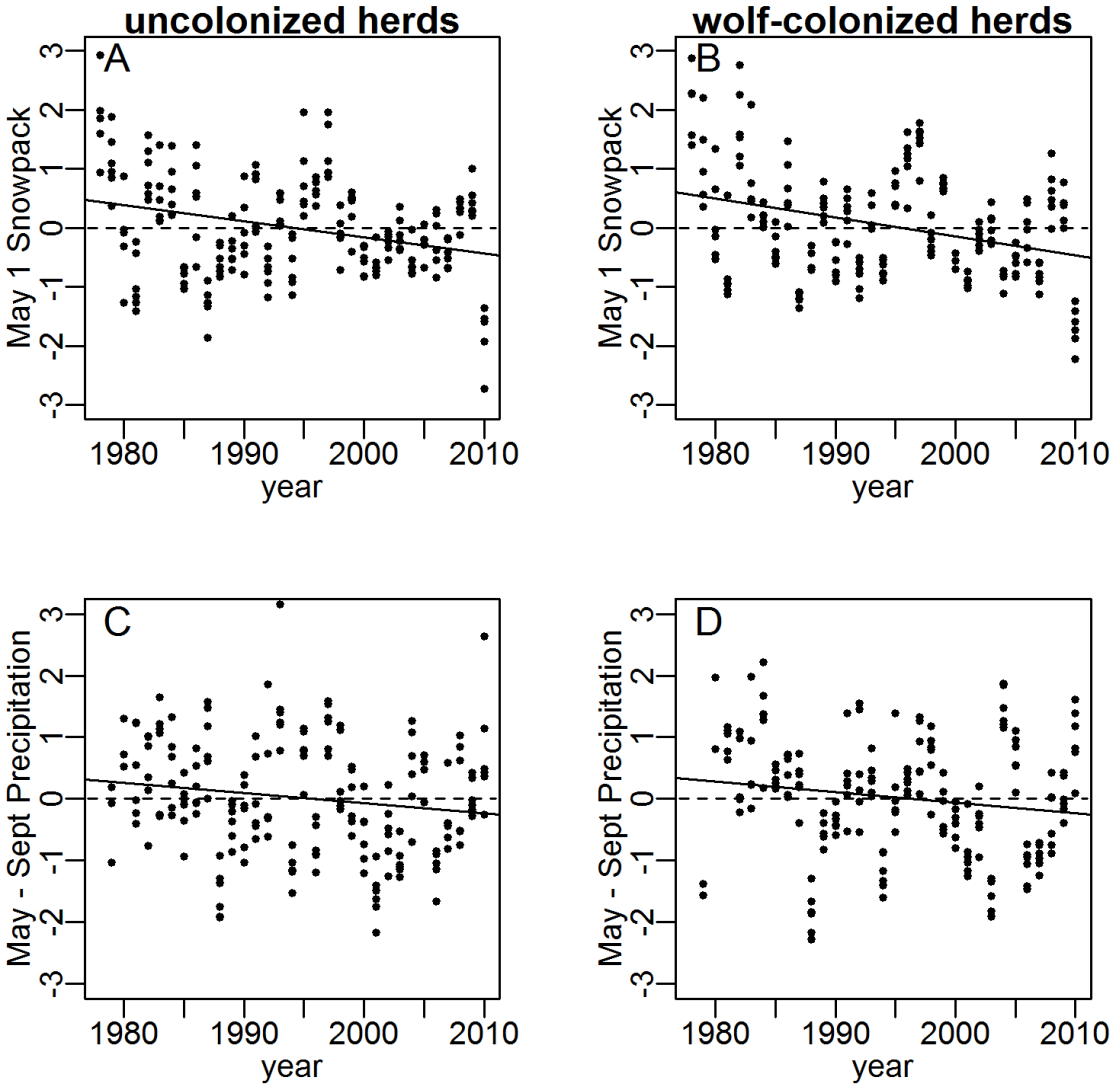
Trend (solid lines) in standardized May 1 snowpack and May through September cumulative precipitation at monitoring stations located within the annual ranges of six elk herds outside and six elk herds within the Greater Yellowstone Area (GYE), 1978–2010.

Weighted-mean coefficients from six linear regressions (one per wolf-colonized herd) of absolute deviations in juvenile∶female ratios from herd-specific means confirmed that elk density (annual winter counts) and late-winter snowpack (May 1 snowpack at SNOTEL sites) had negative effects on juvenile∶female ratios before wolf reintroduction (Table 1). This result is consistent with the consensus in the pre-wolf literature. We detected no effect of elk density or late-winter snowpack on juvenile∶female ratios in wolf-colonized herds after colonization (Table 1), even though the combined count declined across the six herds from 40 636 to 25 676 individuals (Fig. 1C) and late winter snowpack also declined (Fig. 3).

**Table 1:** Weighted mean regression coefficients and standard errors (P-values) from linear models of recruitment as a function of standardized elk counts and May 1 snowpack in six herds colonized by wolves and six uncolonized herds (cumulative May–September precipitation was excluded from the model fit to estimate other coefficients).

treatment	period	elk count	May 1 snowpack	May–September precipitation
colonized by wolves	pre-wolf 1978:1995^1^	−0.023±0.007 (0.026)	−0.016±0.007 (0.086)	−0.001±0.007 (0.940)
	post-wolf 1996:2010	0.011±0.004 (0.029)	0.002±0.003 (0.539)	−0.002±0.006 (0.733)
	full series 1978:2010	0.013±0.005 (0.050)	0.006±0.010 (0.564)	0.004±0.007 (0.552)
uncolonized by wolves	1978:1995	−0.011±0.009 (0.279)	−0.019±0.007 (0.032)	−0.011±0.008 (0.202)
	1996:2010	0.030±0.016 (0.117)	−0.018±0.008 (0.082)	0.003±0.012 (0.804)
	full series 1978:2010	−0.010±0.005 (0.079)	−0.203±0.003 (0.002)	−0.000±0.010 (0.964)

1 Includes one post-wolf year, 1996, for a significantly better fit. Coefficients without this year: count −0.019±0.008, P = 0.062; snow −0.010±0.010, P = 0.368; intercept 0.050±0.010, P = 0.003.

Herds that were never colonized by wolves experienced declines in late winter snowpack that were very similar to those observed for colonized herds (Fig. 3). For uncolonized herds, weighted mean regression coefficients showed that the negative effects of late-winter snowpack on recruitment did not lessen in the post-wolf period (Table 1) as seen in colonized herds. We detected no effect of elk numbers on juvenile∶female ratios in herds uncolonized by wolves in either time period. A trend for a negative effect of elk numbers was apparent in the full time period, suggesting negative density dependence was operating but only detectable over the full range of densities observed (Table 1). A priori, strong negative density-dependent effects in uncolonized herds were less likely given that elk counts (and densities) were roughly one-third of counts in wolf-colonized herds (Fig. 1).

We found no evidence that cumulative growing season precipitation (May 1–September 30 cumulative precipitation at SNOTEL sites) played a role in recent declines in juvenile∶female ratios in wolf-colonized herds. Growing season precipitation declined in uncolonized herds after wolf reintroduction (−0.330 SD's, 95% CI: −0.167, −0.492, P<0.001) but not in colonized herds (−0.078 SD's, 95% CI: 0.077, −0.234, P = 0.323, Fig. 3). These patterns run opposite to the prediction from the hypothesis that growing-season precipitation can explain the observed changes in elk recruitment and dynamics. Growing-season precipitation had no detectable effect in linear models of juvenile∶female ratios in wolf-colonized herds fit to pre-wolf, post-wolf, or the full 33-year data sets (Table 1), and we excluded precipitation from further analysis.

### Observed versus expected recruitment after wolf reintroduction using linear modelling

**Figure 4:**
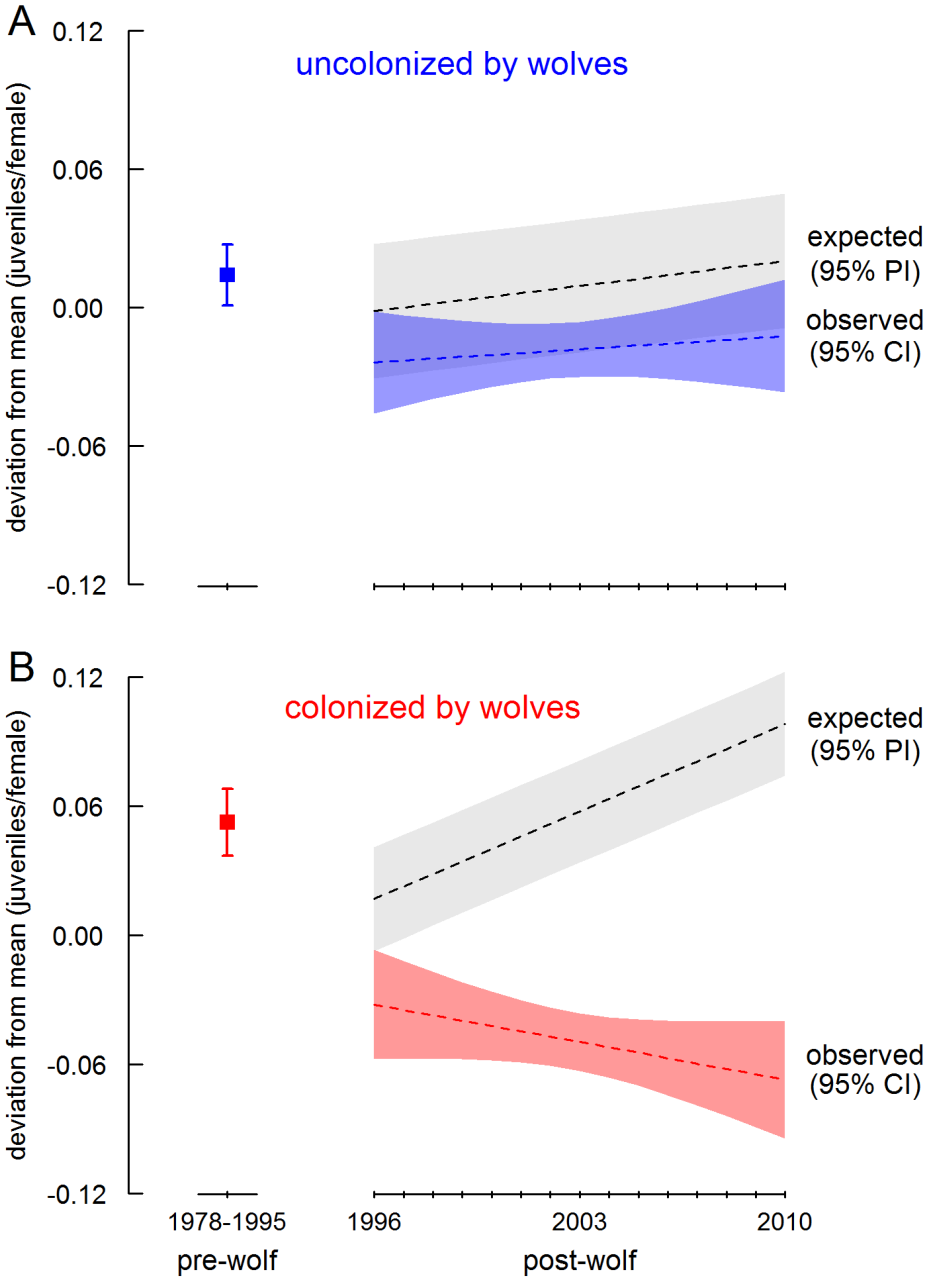
Observed and expected recruitment trends in elk herds (A) uncolonized and (B) colonized by wolves, expressed as deviations from 33-year (1978–2010), herd-specific means. Trends in expected recruitment (±90% prediction intervals) are from predicted responses to the observed winter severity and elk density in post-wolf years (1996–2010) using coefficients from a linear model fit with pre-wolf (1978–1996) data. Pre-wolf means (whiskers, 95% CI) are also shown. In wolf-colonized herds, recruitment was expected to increase dramatically due to declining snowpack and elk density.

In wolf-colonized herds, juvenile∶female ratios were relatively low immediately prior to wolf reintroduction (Fig. 4B), likely because of strong negative density-dependent effects associated with high elk densities (Fig. 1, Table 1). Weighted mean regression coefficients fit using *pre-wolf* data (Table 1) applied to the observed elk numbers and late winter snowpack in *post-wolf* years predicted juvenile∶female ratios would increase after wolf reintroduction due to declining snowpack (Fig. 3) and declining elk numbers (Fig. 1). Consequently, expected mean juvenile∶female ratios for the post-wolf period (0.299 juveniles/female ±0.104, 95% PI) were nearly identical to pre-wolf ratios (as elk density fell through the same range of densities through which it grew prior to wolf colonization). However, the observed post-wolf juvenile∶female ratios were 35% lower than expected on the basis of density and snowpack. The mean difference between observed and expected ratios in the post-wolf period was −0.106 juveniles/female (±0.022, 95% CI, Fig. 4B), comparable to the mean difference of −0.104 juveniles/female between pre-wolf and post-wolf periods in colonized herds.

In herds uncolonized by wolves, late winter snowpack declined by an amount similar to that found in herds colonized by wolves (Fig. 3), so juvenile∶female ratios were expected to trend upwards after wolf reintroduction, as observed (Fig. 4A). Mean juvenile∶female ratios were 7% lower than predicted by our linear models for herds uncolonized by wolves (elevation of trend lines, Fig. 3A). However, uncolonized elk herds were at relatively low densities in the pre-wolf period (compare scales of y-axes in Figs. 1B and 1C) and elk numbers increased after wolf reintroduction in contrast to herds colonized by wolves, which declined (Figs. 1B and 1C). Because the observed values for elk density after wolf reintroduction lie considerably outside the data used to fit regression coefficients for the predictive model, the 7% difference between observed and expected juvenile∶female ratios in uncolonized herds should be interpreted cautiously but it is consistent with a non-linear negative density-dependent effect as have been described previously in elk [11], [37], and is one logical explanation, consistent with our other results, for why juvenile∶female ratios in uncolonized herds were less than predicted by a model fit to data with lower elk densities. (Note that this issue does not pertain to wolf-colonized herds, for which mean density rose and fell through similar densities in the pre-wolf and post-wolf periods (Fig. 1B)).

### Estimating the effect of direct wolf predation on post-wolf juvenile∶female ratios

Observed kill rates of juvenile elk in wolf-colonized herds [35] estimated that each wolf killed 6.45 juvenile elk before the mean date (± SD) of midwinter surveys (3 March ±31.5 d). Incorporating the actual date of midwinter surveys and number of wolves in each herd-year, estimated total annual offtake across the six colonized herds was 193 juveniles in 1996 (by the original 31 introduced wolves) and increased steadily to a peak of 1 855 juveniles in 2007 (by 275 wolves, Fig. 1), after which time wolf numbers and offtake declined. Estimated total mean offtake was 1 151 (±495 SD) juveniles/year since reintroduction, or 0.246 (±0.070, 95% CI) of the estimated total juveniles in herd counts in the same period (0.197 if predated juveniles are included with counts). Alternately, mean offtake of juveniles can be restated as 0.054 (±0.014, 95% CI) juveniles∶counted female (i.e., an average of 1 151 juveniles killed per 21 088 females counted annually). This rate of wolf predation accounts for 52% of the observed decrease (0.104 juvenile/female in midwinter, i.e., 0.054/0.104 = 52%). To summarize, only 1 151 of the mean 2 193 juveniles ‘missing’ annually from herd counts since reintroduction could have been juveniles killed by wolves before they could be counted.

Thus, after considering effects of harvest, growing season precipitation, density dependence, winter severity, and wolf offtake, nearly half of the decline in juveniles/female since reintroduction remains unexplained.

## Discussion

**Figure 5:**
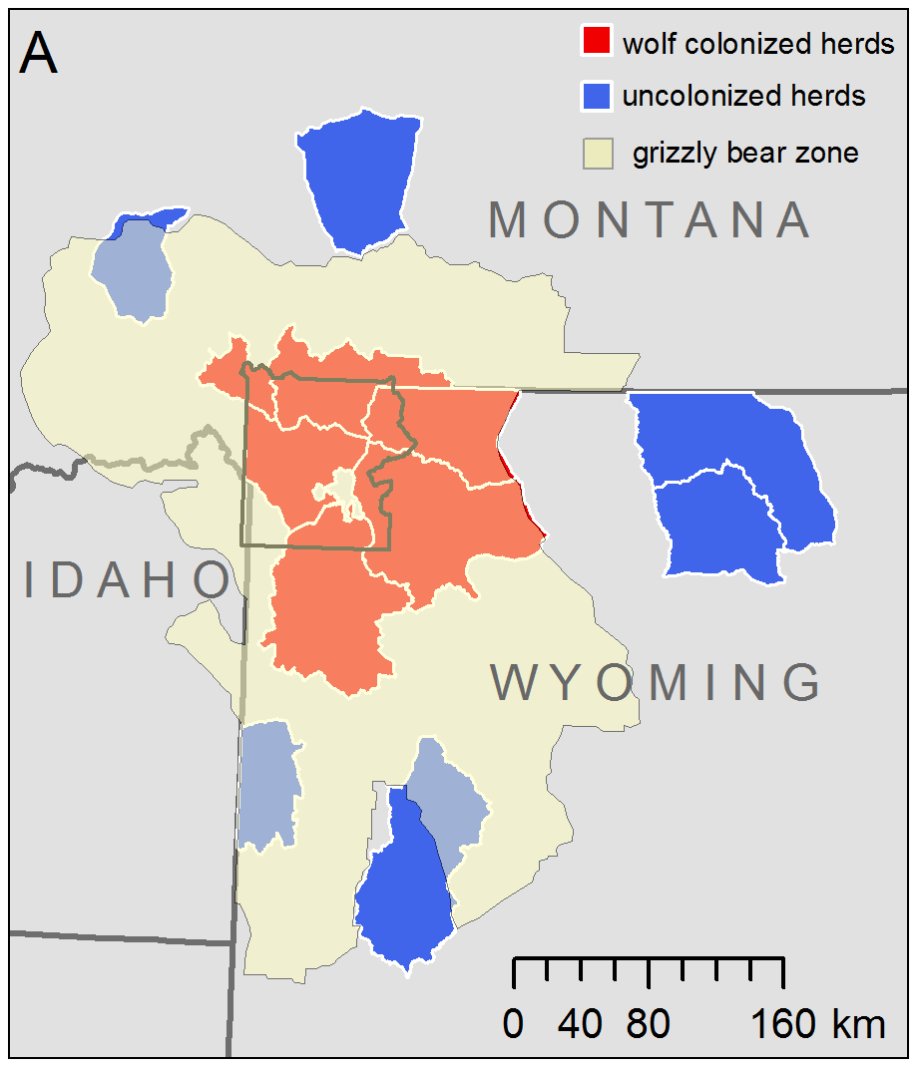
Overlap between the annual ranges of the12 elk herds in this study and the current region within which sightings and mortalities of grizzly bears are collected by the Interagency Grizzly Bear Study Team (U.S. Geological Survey, Bozeman, Montana, USA) for monitoring recovery of grizzly bears. Note that regions between the 12 elk herds considered in this study contain additional elk herds that were not considered by this study but were also exposed to wolf and bear predation.

Large declines in midwinter juvenile∶female ratios have previously been reported from several elk populations in the GYE [15], [20], [29]. Our results show that this phenomenon is largely restricted to the range of the recovered wolf population and correlates with regional declines in elk abundance (Fig. 1). Much smaller declines in juvenile∶female ratios in elk herds not colonized by wolves were not detectable at the herd level and consistent with non-linear density-dependent effects in these populations, which continued to grow [11], [37]. The small decrease in recruitment outside the wolf recolonization zone might also be explained by other factors such as changes in relative harvest rates of juveniles (see Results) or perhaps, decreased survival due to an expanding grizzly bear population [38], [39]; (see Fig. 5 and below for further discussion of regional grizzly bear population trends).

Juvenile∶female ratios in uncolonized elk populations suggest that the effects of negative density dependence and winter severity continue to operate on elk dynamics, but these formerly dominant forces may now be less conspicuous alongside the effects of wolf reintroduction [19]. The mean decline in juvenile∶female ratios (35%) between colonization periods, matched by a 37% decline in harvest ratios, was equal to the difference between observed and expected ratios after accounting for the effects of declining density and snowpack (35%) largely because mean elk densities were comparable across the two periods (rising through those densities in the pre-wolf period and falling through them in the post-wolf period: Fig. 1). Declining elk density and snowpack covaried with wolf reintroduction in these data, and disentangling these effects was not entirely possible without a truly randomized experiment, but it is unlikely that these two well-established effects on elk recruitment no longer operated in wolf-colonized herds, particularly because they continued to operate in uncolonized herds (Table 1). What seems more likely is that positive effects of declining density and snowpack on recruitment in recent years have been outweighed by a larger negative effect of wolf reintroduction.

We found that wolf offtake of juvenile elk could explain only half the observed decline in midwinter juvenile∶female ratios. Mean kill rates could potentially underestimate offtake in some herds, but Metz et al's [35] estimates are derived from data collected 1995–2009 from the study area itself, perfectly overlapping the post-wolf period in this study. Further, Metz et al's [35] mean kill rates include seasons when kill rates on juveniles were highest (post-partum) and herd-years where wolf∶elk ratios were exceptionally low (i.e. immediately after wolf reintroduction) producing some of the highest kill rates reported for wolves [40]. This effect is most evident in the Madison-Firehole elk herd in central Yellowstone where observed elk kill rates were lower and wolves also preyed on bison (*Bison bison*) [34]: Metz et al's [35] kill rates applied to this herd estimated mean annual juvenile offtake equivalent to 123% of the mean population size [29]. Thus, it is unlikely that our methods underestimated wolf predation rates or their contribution to declining midwinter juvenile∶female ratios However, several additional sources of uncertainty accompany the estimated contribution of wolf predation to midwinter juvenile∶female ratios, and three primary factors with supporting evidence are worth noting:

1. Sightability of elk during herd counts is <1. Our estimate for the contribution of wolf predation rate expresses offtake as a proportion of uncorrected herd counts. Sightability of elk during herd counts on Yellowstone's relatively open northern winter range varies between 0.50 and 0.83 [9], [10] and is considerably less than this in more forested areas, depending on group size [41]. Inflation factors of 1.32 to 1.41 have been applied to some of these same herd counts to estimate the predation rate of wolves [19]. If we applied similar inflation factors to account for sightability, our estimates of the proportion of missing calves that can be attributed to direct predation would decrease accordingly, leaving an even larger proportion of the recruitment decline unexplained.
2. The relative abundance of adult females has changed between the pre-wolf and post-wolf periods. Wolves now kill 1 female elk for every 1.3–3.8 juveniles prior to the midwinter classification surveys [35], [40] and hunters harvested 7.3 females per juvenile in the post-wolf period versus 4.6 prior to wolf reintroduction (see Results). Annual female survival in ‘prime-age’ animals in northern Yellowstone was 0.99 prior to wolf reintroduction but has subsequently decreased by 14% to 19% [15], [22]. Declining female survival has been seen in additional GYE elk herds, but not in herds uncolonized by wolves [26], [42]. If we considered declining abundance in both the numerator *and* denominator in the juvenile∶female ratio, the expected influence of direct wolf predation on midwinter juvenile∶female ratios would be lessened.
3. Predation on juvenile elk does not cause fully additive effects on juvenile survival. In GYE elk, Barber-Meyer et al. (2008) found only 5% of juvenile mortalities were caused by disease, starvation or exposure following wolf reintroduction versus 45% prior to wolves as seen by Singer et al (1997). Garrott et al. (2009) noted that 93% of juvenile elk mortality on their study site in central Yellowstone was attributed to starvation prior to wolves, versus 17% of juvenile mortality after wolf reintroduction. Griffin et al (2011) compiled fates from 1,999 marked juvenile elk in 12 populations (including the GYE) and found no evidence for an additive effect of wolf predation on juvenile survival. We know of no study with evidence for strong additive effects of wolf predation on juvenile elk survival, but additivity may be difficult to detect because juvenile mortality due to wolf predation is consistently low (e.g., averaging 4% in Griffin et al. [2011]). Nevertheless, any degree of compensation would decrease the proportion of missing calves that can be explained by observed rates of wolf predation. Here again, this consideration suggests that less than half of the decline in recruitment can be explained by direct wolf predation in combination with other established drivers of elk dynamics.

To be clear, wolves kill a large number of juvenile elk annually in the GYE, but the magnitude of this offtake relative to the prey base suggests that direct killing by wolves has less influence on midwinter juvenile∶female ratios than other limiting factors. Additional offtake from increased grizzly bear predation on juveniles since wolf reintroduction has been hypothesized as an alternative explanation for declining recruitment [39] as grizzly bears can be heavy predators of juveniles in the first 30 days of life, although bears rarely kill elk outside this period [20], [38], [43]. There is evidence that range expansion and increasing bear density on the periphery of the grizzly's range have occurred since wolf reintroduction [44], [45]. These changes might have contributed to localized declines in juvenile survival in some areas [39], including some of the uncolonized herds in this study (Fig. 5).

Published data on spatial distribution of grizzly bears in this ecosystem were not available. However, the regional grizzly bear population, and its trend, have been closely monitored since listing as a threatened species in 1975, and are sufficient for a broad test whether increased grizzly bear predation can quantitatively account for the observed changes in elk recruitment. After wolf reintroduction, Yellowstone grizzly bears were found to kill one juvenile elk/bear/4.3 days over the first 30 days of a juveniles' life [46] and bears rarely killed elk outside this period [10], [20], [38], [43]. Thus, approximately 7.0 juveniles/bear are killed prior to midwinter classification surveys (but note, only by those bears that spatially overlap neonatal elk distributions in June, [20], [39], [46]). This predation rate is comparable to that of wolves, which implies that grizzly bears must have undergone comparably rapid population growth after wolf reintroduction if increased bear offtake can explain a substantial portion of the decline in elk recruitment. For example, for the annual average of 1 042 juveniles missing from counts that cannot be explained by wolf offtake, elk density, or winter severity (2 193–1 151), a mean increase of 149 adult bears since wolf reintroduction (1 042 juveniles/7.0 juveniles/bear/year) would explain the otherwise unexplained remainder of the midwinter decline in the juvenile∶female ratio (ignoring the implications of sightability, declining, female survival and compensatory mortality discussed above). An additional 149 bears in the mean post-wolf grizzly bear population, restricted to the parturition areas of our six colonized herds would imply a 1.74-fold increase (geometric mean λ over 1996–2010 of 1.07) in the 1995–1996 population estimate for the entire grizzly bear population in southwestern Montana, northwestern Wyoming and eastern Idaho [44], a region that includes not only the wolf-colonized elk herds in this study, but all or part of several uncolonized elk herds, and several additional elk herds not considered (Fig. 5). This hypothesis can be strongly rejected: grizzly bear density in YNP was directly measured over the interval spanning wolf reintroduction and was stable with a growth rate (λ)indistinguishable from one with sufficient power to detect bear population increases far smaller than 174% [47]. While not completely overlapping the annual ranges of the wolf-colonized herds in this study, YNP largely corresponds to the parturition and summer ranges of most of the migratory, wolf-colonized elk in this study where neonatal elk densities are high [20], [33], [46], [48]. The reported magnitude and spatial patterns of grizzly bear population growth [45], [49] and predation rate [50] provide evidence contrary to the hypothesis that increased grizzly bear predation can explain the observed pattern of regional declines in elk recruitment since wolf reintroduction.

The finding that wolf offtake explains only half of the observed decline in uncorrected herd counts is not a new result and aligns well with previous research that has inferred that wolf predation rates (combined with other drivers such as density and snowpack) cannot fully explain the observed declines in elk abundance [18], [19], [22], [23] or juvenile survival [20], [43]. However, direct predation is not the only mechanism by which predators influence prey demography and population dynamics. Risk effects (or non-consumptive effects) on prey populations arise when responses to predation risk carry costs to survival and reproduction. Risk effects have been found in a wide array of predator-prey systems [51]–[53], and should be strong in long-lived, highly iteroparous species that produce only one juvenile per year, such as elk. In wolves and elk, five studies spanning 10 elk herds reported 24–43% declines in elk pregnancy rates following increased predation risk (summarized by Creel et al. 2011). For most of these herds, the pregnancy rates were measured with standardized methods and systematically stratified sampling over the same time period each winter, over multiple years (Creel et al. 2007). One additional study not discussed by Creel et al (2011) also reported pregnancy rates in Yellowstone females across “prime-age” (2.5 to 9.5 years) age classes that averaged 0.696 in the first 5 years after wolf reintroduction [23], a 27% decline from the 0.953 pregnancy rate reported for the same herd over several years in the 1960's [54]. Declining pregnancy rates would produce a direct, one-to-one decline in juvenile∶female ratios that should be conspicuous throughout the year, but little data exist on herd demography outside of winter. Where such data do exist, lower summer and fall juvenile∶female ratios after wolf recolonization have been found in several herds [55], [56] while we found autumn harvest ratios of juveniles∶female declined by 37% in wolf-colonized herds but not in uncolonized herds (see Results). These data strongly suggests that pregnancy declines of 24–43% have translated into comparable 35% declines in recruitment leading to negative population growth (Fig. 2).

The risk effects hypothesis, that predation risk from wolves may be contributing to population declines in their primary prey via behavioral, nutritional, and physiological pathways, has been challenged by others [57]–[59]. After wolf reintroduction, strong behavioral and nutritional responses to predation risk from wolves were detected [60]–[63], together with declines in survival and fecundity too large to be explained by wolf predation alone [16], [43], [64], [65]. Middleton et al. [59] suggested that behavioral responses to predation risk were too infrequent and weak to cause demographic costs, but their methods only measured responses to wolves that carried GPS collars (ignoring uncollared wolves), and only at the times that these collars recorded locations. Such methods greatly underestimate the frequency and strength of antipredator responses [66], and direct observations of wolf presence are much higher [67]. Despite this problem, Middleton et al. [59] detected effects of wolf presence on elk behavior and movements, and pregnancy rates were lower in the presence of wolves in Middleton's data, as in other GYE populations [16].

Both risk effects and direct predation from wolves contribute to the total effect of wolves on elk populations and our results suggest that declines in midwinter juvenile∶female ratios are not well-explained by other factors without risk effects at least as large as the predation rate on juveniles. To our knowledge, risk effects are the only ecological mechanism hypothesized to cause a strong demographic response to a predator when the direct predation rate is relatively low and largely compensatory [51], [53], [56], [68], [69], as has been reported for wolf predation on elk calves [43]. Here, we found that this result arises over a very large spatial scale and a long span of years, after considering all of the other factors demonstrated (or hypothesized) to affect elk recruitment in the GYE. It is no more logical to assume that risk effects are equal to zero than it is to assume that the rate of direct predation is zero, but this assumption is common because risk effects arise through a subtle, integrative process that results in depressed fecundity or survival in much less dramatic fashion than direct predation. Because antipredator behavior and demography vary on different time scales, and demography is affected by many factors other than predation risk, it is exceptionally difficult to gather data that can detect a relationship between the two but increasingly, approaches like the one used here are suggesting that the relationship may often be important [68], [70].
